# Antibody dual-functionalisation enabled through a modular divinylpyrimidine disulfide rebridging strategy†

**DOI:** 10.1039/d2cc02515a

**Published:** 2022-07-22

**Authors:** Abigail R. Hanby, Stephen J. Walsh, Andrew J. Counsell, Nicola Ashman, Kim T. Mortensen, Jason S. Carroll, David R. Spring

**Affiliations:** Yusuf Hamied Department of Chemistry, University of Cambridge Lensfield Road Cambridge CB2 1EW UK spring@ch.cam.ac.uk; Cancer Research UK Cambridge Institute, University of Cambridge Robinson Way Cambridge CB2 0RE UK

## Abstract

Herein we report the development of a methodology for the dual-functionalisation of IgG antibodies. This is accomplished through the combination of disulfide rebridging divinylpyrimidine technology, with bicyclononyne and methylcyclopropene handles to facilitate sequential SPAAC and IEDDA reactions. Advantageously, the strategy does not require metal catalysis and avoids the need for purification between functionalisation steps.

## Abstract 2

A modular and metal-free chemical methodology for the synthesis of dual-functionalised antibody-drug conjugates.

## Graphical abstract

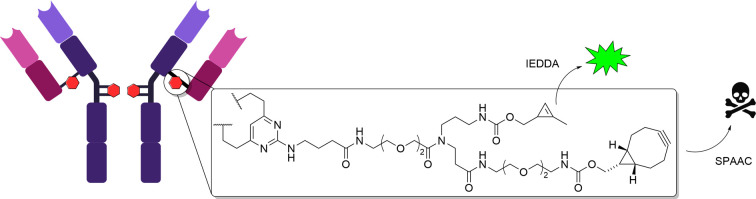

Antibody-drug conjugates (ADC) are a class of therapeutic that has experienced rapid growth in recent times, with the US Food and Drug Administration having granted marketing approval to eleven thus far. Their remarkable therapeutic efficacy is primarily attained through combining the high target selectivity of monoclonal antibodies, with highly potent small molecule payloads. These components are tethered *via* a chemical linker, which often facilitates a mechanism for enzymatic payload release, in addition to modulation of the pharmacokinetic properties of the therapeutic. Heterobifunctional conjugates offer potential routes to the improvement of therapeutic efficacy, to the mitigation of acquired drug-resistance through multi-drug ADCs, or towards the synthesis of targeted theranostics.^1,2^ However, most linkers are designed to accommodate a single payload, which either limits or complicates their use in the development of conjugates with dual-functionalisation. Approaches towards dual-functionalised conjugates typically employ a combination of distinct conjugation techniques,^3–5^ genetic incorporation of orthogonal unnatural amino acids,^6^ linkers with pre-existing dual-functionalisation,^7^ or a multifunctional linker which facilitates bioorthogonal reactions.^8–10^ While many of the current approaches are effective, they tend to be cumbersome and the majority rely on the use of cytotoxic metal catalysts (*viz.* copper-catalysed azide-alkyne cycloadditions). The latter is particularly problematic due to the potential for residual metal contaminants, and unintended oxidation of amino acid residues,^11,12^ which can result in negative immunogenic responses.

A number of metal-free alternatives for the chemical modification of antibodies have been explored through the use of unnatural amino acids containing reactive moieties such as cyclopropene,^13^ azide,^14^ and bicyclononyne.^15^ However, the use of these techniques has largely been limited to the mono-functionalisation of antibodies. In one of the few dual-functionalisation reports, Walker *et al.* utilised transglutaminase-mediated ligation (mTG) to attach a multifunctional linker, which enabled a strain-promoted azide-alkyne cycloaddition (SPAAC) followed by inverse-electron-demand Diels–Alder reaction (IEDDA), in order to generate a bioconjugate containing FRET-paired fluorophores, and a PEGylated ADC.^16^ Similarly, Yamazaki *et al.* have reported the synthesis of dual-drug ADCs with an azide/tetrazine linker installed at Q295 by mTG.^17^ As demand for multifunctional conjugates increases, there is a growing need for scaffolds which offer greater flexibility and versatility to enable rapid exploration of payload-antibody combinations. Accordingly, we sought to develop the divinylpyrimidine (DVP) disulfide rebridging linkers 1–3 (Fig. 1), which could facilitate either mono- or dual-functionalisation of off-the-shelf antibodies in a metal-free and bioorthogonal fashion. It was anticipated that this approach would benefit from the high conversion, stability, and site-selectivity observed with DVP reagents,^18–20^ whilst the use of bioorthogonal SPAAC and IEDDA reactions would allow for modular and late-stage functionalisation of the antibody. Furthermore, prudent selection of strained alkyne and dienophile reactants would facilitate the proposed sequential SPAAC and IEDDA reactions, whilst avoiding the need for stepwise purification. Of further note regarding the linker design is the modest spacing between the DVP motif and the attachment point(s), in order to improve accessibility for modification following antibody conjugation.

**Fig. 1:**
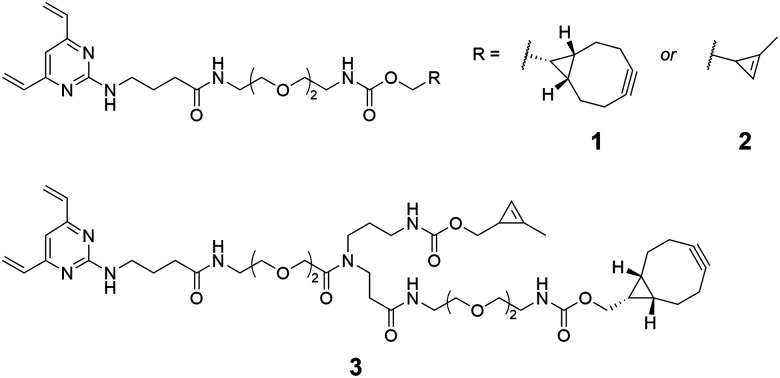
Bioorthogonal monofunctional (1 and 2) and dual-functional (3) divinylpyrimidine linkers synthesised in the present study.

It was considered expedient to focus the initial investigation on the mono-functionalised DVPs 1 and 2. Accordingly, both linkers were synthesised in four steps from 2,4,6-trichloropyrimidine (see ESI†). The anti-HER2 IgG trastuzumab (Herceptin) was selected as an appropriate antibody with which to undertake validation of the rebridging efficiency of the synthesised DVPs, on the basis of its successful use in the treatment of HER2-positive breast cancer, incorporation in multiple FDA-approved ADCs, and its widespread use in bioconjugation method development.

TCEP-mediated reduction of the four interchain disulfides, followed by treatment with 20 equivalents (5 per disulfide) of DVP 1 or 2 (Fig. 2), was observed by LCMS and SDS-PAGE analysis to produce the rebridged trastuzumab conjugates 4 and 5 in >95% conversion (Fig. 2). As has been observed for other disulfide rebridging reagents, the predominant conjugate species observable by SDS-PAGE is that of the intrachain re-bridged ‘half antibody’.^18,21^ Investigation of the relationship between conditions and rebridging efficiency was briefly undertaken with DVP 1, which revealed no significant improvement in rebridging efficiency beyond 2 hours of reaction time, nor with a greater excess of linker (Table S1 and Fig. S1, ESI†).

**Fig. 2:**
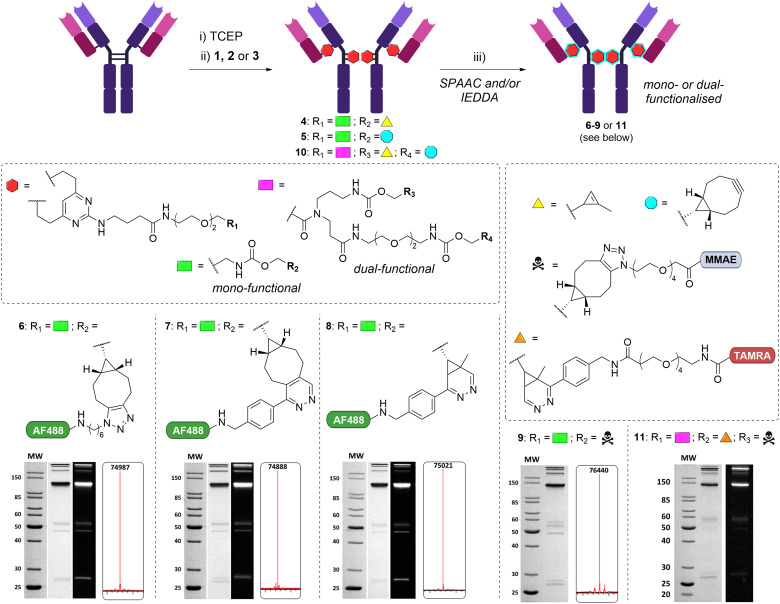
Interchain disulfide rebridging of trastuzumab with monofunctional DVP 1 or 2 or dual-functional DVP 3, yielded antibody conjugates 4, 5 or 10. SPAAC and/or IEDDA with AlexaFluor488-azide, AZDye488-tetrazine, or N_3_-PEG_4_-VC-PAB-MMAE facilitated conversion to antibody conjugates 6–9 and 11. Regents and conditions: (i) TCEP (10 equiv.), 25 mM tris HCl 25 mM NaCl, 0.5 mM EDTA (TBS) pH 8, 37 °C, 1 h; (ii) DVP 1, 2 or 3 (20 equiv.), 10% DMSO in TBS pH 8, 37 °C, 2 h; (iii) 10% DMSO in PBS, 37 °C and in the case of 6: AlexaFluor488-Azide (16 equiv.), 8 h; 7–8: AZDye488-Tetrazine (16 equiv.), 1 h; 9: N_3_-PEG_4_-VC-PAB-MMAE (16 equiv.), 8 h; 11: N_3_-PEG_4_-VC-PAB-MMAE (24 equiv.), 16 h, followed by TAMRA-tetrazine (24 equiv.), 4 h. SDS-PAGE (12%, reducing conditions) of in-gel fluorescence (6–8 and 11) and Coomassie stain shown for 6–9 and 11. Deconvoluted mass spectrum shown for 6–9. See ESI† for full details.

Following on from the successful rebridging of trastuzumab, a series of post-rebridging modifications were undertaken to demonstrate the amenability of the conjugate to further functionalisation. Commercially available AlexaFluor488-Azide (ThermoFisher) and AZDye488-Tetrazine (ClickChemistryTools) were selected as fluorescent payloads. Conjugate 4 was incubated with 16 equivalents of AlexaFluor488-Azide at 37 °C in PBS (10% DMSO) for 8 hours. SDS-PAGE confirmed fluorescent labelling of the antibody and LCMS analysis confirmed production of the desired conjugate 6. UV-vis spectroscopy revealed the fluorophore-to-antibody ratio to be 4.0. Likewise, conjugates 7 and 8 were synthesised *via* a SPAAC from 4 and 5 respectively, with complete conversion observed with 16 equivalents of AZDye488-Tetrazine, and FARs determined with UV-vis to be 4.1 and 4.0 (Fig. S8, ESI†).

To demonstrate wider applicability of the platform, it was decided to incorporate the well-validated and highly-potent tubulin inhibitor monomethyl auristatin E (MMAE). Multiple FDA-approved ADCs incorporate a protease-labile Val-Cit-PAB-MMAE motif for efficient release of the free drug following cellular internalisation. Attachment of this motif to conjugate 4 was found to be possible. The reagent N_3_-PEG_4_-val-cit-PAB-MMAE was synthesised (ESI†) and utilised in 16 equivalents at 37 °C for 8 hours to generate model ADC 9. Excellent attachment of the MMAE payload was confirmed by LCMS analysis of conjugate 9.

Having successfully explored the mono-functionalisation of trastuzumab with DVPs 1 and 2, efforts towards a linker comprising both the methylcyclopropene and BCN groups could commence. Synthesis of the dual functional DVP linker 3 was achieved in 7 steps (see ESI†). Reduced trastuzumab was treated with 20 equivalents of DVP 3 at 37 °C for 2 hours. SDS-PAGE and LCMS analyses confirmed in excess of 90% rebridging to form the desired conjugate 10 (Fig. 2).

To illustrate the utility of the orthogonally functionalised linker, a theranostic ADC comprising enzyme-cleavable MMAE and a fluorescent moiety was designed. The N_3_-PEG_4_-Val-Cit-PAB-MMAE reagent previously synthesised was used for attachment of the drug. It was reasoned that attachment of a second payload to the linker may be more difficult than the first due to steric effects, and so it was proposed to opt for commercially available TAMRA-tetrazine (ClickChemistryTools), which possessed a PEG_4_ spacing motif between the attachment handle and the dye. Gratifyingly, upon subjection of intermediate 10 to SPAAC with 24 equivalents of the MMAE reagent at 37 °C for 16 h, followed by IEDDA with 24 equivalents of TAMRA-tetrazine under the same conditions, one-pot conversion to ADC 11 was confirmed by SDS-PAGE and LCMS analysis. The versatility of DVP 3 toward attachment of different payloads was also exemplified by conjugation of a sulfatase-cleavable MMAE payload to intermediate 10, and AZDye488 (ESI†). Again, formation of the desired conjugate was achieved without the need for stepwise purification.

To demonstrate the ability of the fluorescent ADC 11 to facilitate observation of ADC uptake, we incubated 11 with HER2-positive SK-BR3 cells (American Type Culture Collection) and performed live cell microscopy (Fig. 3). With 2 h of incubation, mostly staining of the membrane was observed, however significant internalisation could be visualised after 24 h. As expected, no fluorescent signal was observed on the HER2-negative MCF7 cell line (European Collection of Authenticated Cell Cultures). This data demonstrates the excellent cell-selectivity and cell imaging potential of the present approach.

**Fig. 3:**
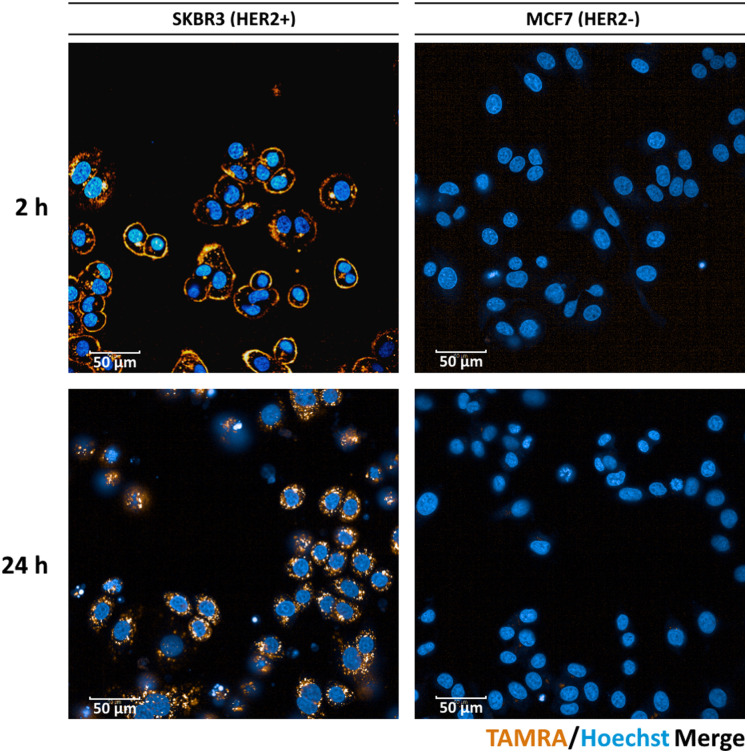
Live cell microscopy images of HER-positive SK-BR3 and HER2-negative MCF7 cells after 2 h and 24 h, following treatment with fluorescent conjugate 11. Scale bar represents 50 μm.

To exemplify the ability of ADCs 9 and 11 to exhibit selective cytotoxicity, cell viability assays were conducted with HER2-positive SK-BR3 and HER2-negative MCF7 cell lines (Fig. 4). MMAE has previously been reported to exhibit sub-nM IC_50_ cytotoxicity against both cell lines.^18^ Pleasingly, ADCs 9 and 11 exhibited IC_50_ values of 26 pM and 40 pM respectively against HER2-positive cell line SK-BR3. No significant cytotoxic activity against HER2-negative MCF7 cells was observed for either ADC, indicating IC_50_ values in excess of 30 nM. The results demonstrate that introduction of the linker scaffold and subsequent dual-functionalisation does not significantly hinder the potency or HER2 selectivity of these ADCs.

**Fig. 4:**
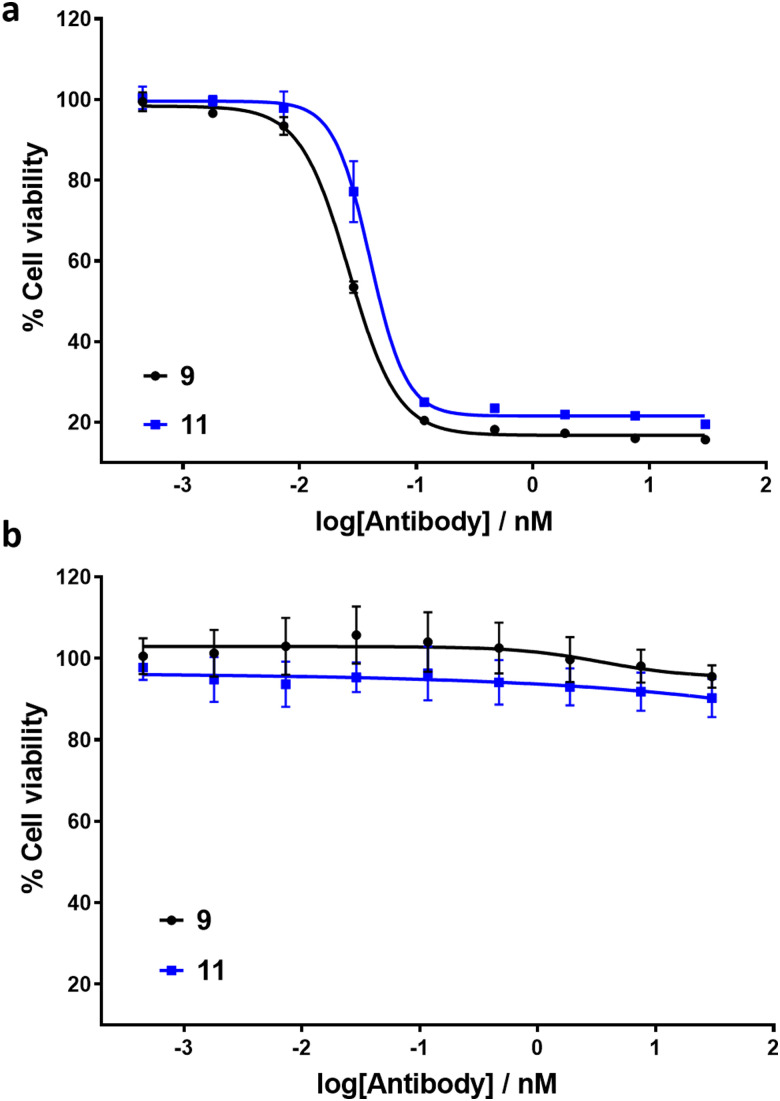
*In vitro* biological evaluation of ADCs 9 and 11 in (a) HER2-positive SK-BR3 cells and (b) HER2-negative MCF7 cells. Viability data shows the mean of three independent experiments and error bars represent the standard error of the mean. IC_50_ values for SK-BR3 cells: ADC 9 = 26 pM; ADC 11 = 40 pM.

We have recently reported the results of a detailed structural characterisation and *in vivo* evaluation of ADCs produced with DVP reagents, which demonstrated their exquisitely selective efficacy and tolerability in breast cancer xenograft models.^22^ The present work builds upon this validation of the use of DVP reagents to develop safe and efficacious ADCs, by introducing additional versatility to the linkage scaffold, facilitating the generation of antibody conjugates with higher orders of functionalisation. Moreover, the work represents the first example of a disulfide rebridging strategy that enables metal-free post-bioconjugation construction of dual-functionalised ADCs. The power of the approach lies in the flexibility of the modular scaffold, and the applicability of the linker to off-the-shelf IgG1s, with no protein engineering required. Each step was demonstrated to proceed in high conversion without the need for arduous purification, and the generated ADCs were shown to exhibit a wide therapeutic window for HER2 across SKBR3 (+ve) and MCF7 (−ve) cell lines. The fluorescent ADC was used to directly observe binding and internalisation of the ADC, and possesses great potential for use in further studies into intracellular trafficking of antibody-payloads.

A. R. H. acknowledges the EPSRC and Cambridge Trusts for funding. A. J. C. acknowledges Trinity College Cambridge for a Krishnan-Ang Studentship. K. T. M. acknowledges the Carlsberg Foundation for funding. The Spring group acknowledges general lab support from the EPSRC, BBSRC, MRC and the Royal Society. Research in the Carroll lab was funded by CRUK core funding awarded to J. S. C.

This work was funded in whole or in part by UKRI grants. For the purpose of Open Access, the author has applied a CC BY public copyright licence to any Author Accepted Manuscript (AAM) version arising.

## Conflicts of interest

There are no conflicts of interest to declare.

## Supplementary Material
